# Conventional Treatment of Maxillary Incisor Type III Dens Invaginatus with Periapical Lesion: A Case Report

**DOI:** 10.5402/2011/257609

**Published:** 2010-10-10

**Authors:** Álvaro Henrique Borges, Alex Semenoff Segundo, Michele Regina Nadalin, Fábio Luís Miranda Pedro, Antônio Miranda da Cruz Filho, Manoel Damião Sousa-Neto

**Affiliations:** ^1^School of Dentistry, University of Ribeirão Preto (UNAERP), 14096-900 Ribeirão Preto, SP, Brazil; ^2^School of Dentistry, University of Cuiabá (UNIC), 78015-480 Cuiabá, MT, Brazil; ^3^School of Dentistry, University of São Paulo (USP), 14040-904 Ribeirão Preto, SP, Brazil

## Abstract

Dens invaginatus is a developmental dental anomaly clinically characterized by a palatine furrow that can be limited to the coronal pulp or may extend to the radicular apex. The purpose of this paper was to present a clinical case of type III *dens invaginatus*, identified on the maxillary right central incisor in anterior periapical radiographs, in which the tooth was submitted to conventional endodontic treatment. The results obtained after five years of clinical and radiographic followup demonstrated that conventional endodontic treatment is a clinically viable alternative in cases of type III dens invaginatus.

## 1. Introduction

Dens invaginatus, also known as pregnant woman anomaly, extensive compound odontoma, and dens in dente, is a developmental anomaly that occurs as a consequence of an invagination on the external surface of the tooth crown before calcification occurs [1–3]. Its etiology is not well understood, yet it is believed that compressed areas in permanent teeth during the formation and eruption process may result in dental crowns with peaks of invaginated enamel in the root canal [4].

Histologically, dens invaginatus can be defined as enamel organ deepening or invagination in dental papilla during the dental organ development process. It begins in the crown and may penetrate throughout the whole root, and it occurs before dental tissue calcification [5–7]. According to Oehlers [8], dens invaginatus can be classified into three categories depending on enamel invagination depth inside the tooth. In type I, the invagination ends as a blind sac and is limited to the coronary portion of the tooth. In type II, the invagination extends beyond the cementoenamel junction and is retained inside the main canal. Type III occurs when the invagination extends throughout the root canal interior and reaches the apical tooth area, giving rise to two or more foramina.

Hovland [2] calculated the dens invaginatus incidence to be from 0.04% to 10.00% for the possibility of occurrence for any tooth, affecting either the deciduous [9] or the permanent dentition [10], and commonly involving the upper lateral incisors [11, 12]. Cases of bilateral occurrence have been reported [13–15]. Therefore, in the event that a tooth is affected, its homologous counterpart will need to be investigated. Rare cases are reported for molars [16], premolars [15], and maxillary central incisors [17]. The cause for the appearance of dens invaginatus is unclear, but some evidence suggests familial and hereditary components [18].

Despite the well-known occurrence of this anomaly, the conservation of teeth bearing this sort of invagination and periapical problems was achieved only in the second half of the last century. Until the 1950s, dens invaginatus that presented pulpal and periodontal problems or apical lesions is condemned to avulsion [13, 19, 20]. The conventional endodontic treatment has been tried with success by several professionals [2, 21, 22].

Types I and II dens invaginatus do not present treatment problems. It is only necessary to remove the invagination, create a tooth with a single canal, and use conventional endodontic treatment [7, 23–25]. For cases of type III, invagination presents communication with the oral cavity [6, 23, 24]. Pulpal tissue invasion by irritants, such as microorganisms, can frequently result in pulp necrosis and periapical lesion development [26]. Several dens invaginatus treatment techniques have been reported. Some authors have described nonsurgical treatments [7, 23, 27–30]; however, there are also periodontal surgery case reports [31–35], intentional reimplantation [36], and removal of the invaginated portion [17].

A literature review was performed using a Medline electronic search, based on case reports about dens invaginatus. The Medline search identified 95 papers published from May 1997 to August 2009. Initially, all abstracts were read to identify papers that fit the requirements for this review: tooth, classification, and treatment (surgical or nonsurgical). The review was undertaken to scrutinize publications dealing with these categories, and it was observed that maxillary lateral incisors were the most affected teeth. Of the nine maxillary incisor cases found, only five noted a description of treatment [37–41]. Type III was more frequently described, and orthograde treatment was cited more than surgical procedures. Thus, the objective of the present study was to report a clinical case of type III maxillary incisor dens invaginatus that was treated with a conventional treatment.

## 2. Case Report

**Figure 1:**
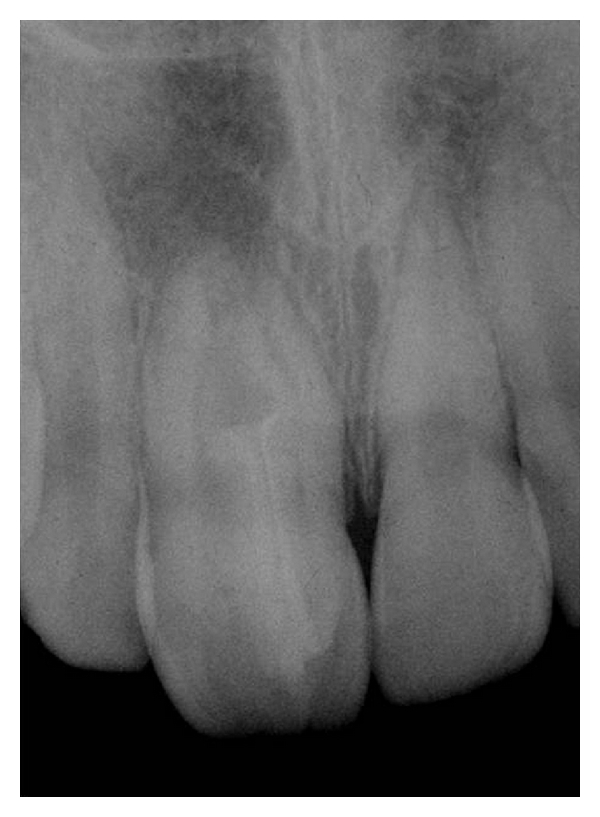
Diagnostic radiograph. Note the presence of type III dens invaginatus and a radiolucid image at the apex of tooth no. 11.

**Figure 2:**
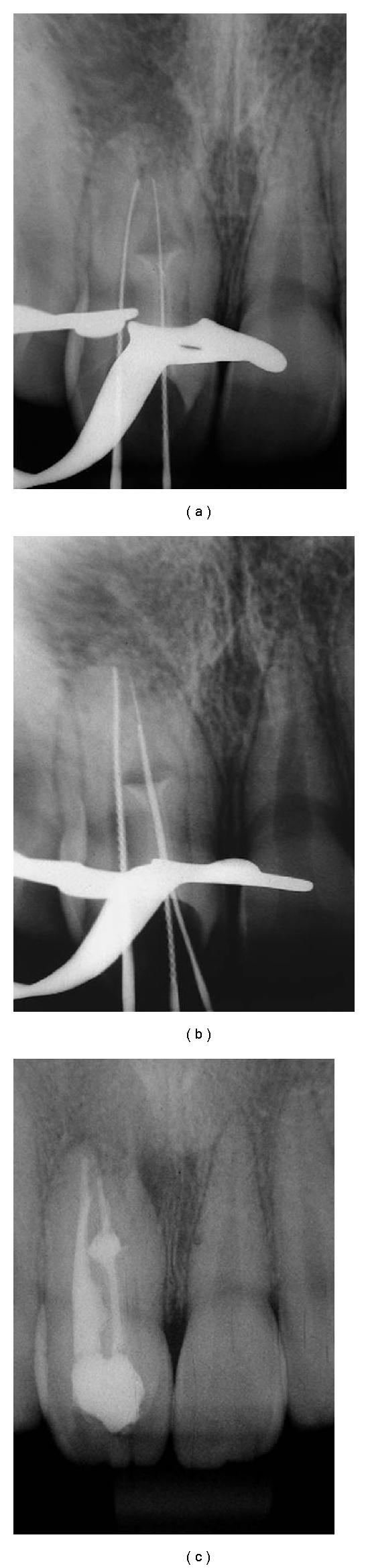
Endodontic treatment. (a) and (b) The work lengths of the two canals were established at one millimeter from the radiographic apex. (c) Root canal filled.

**Figure 3:**
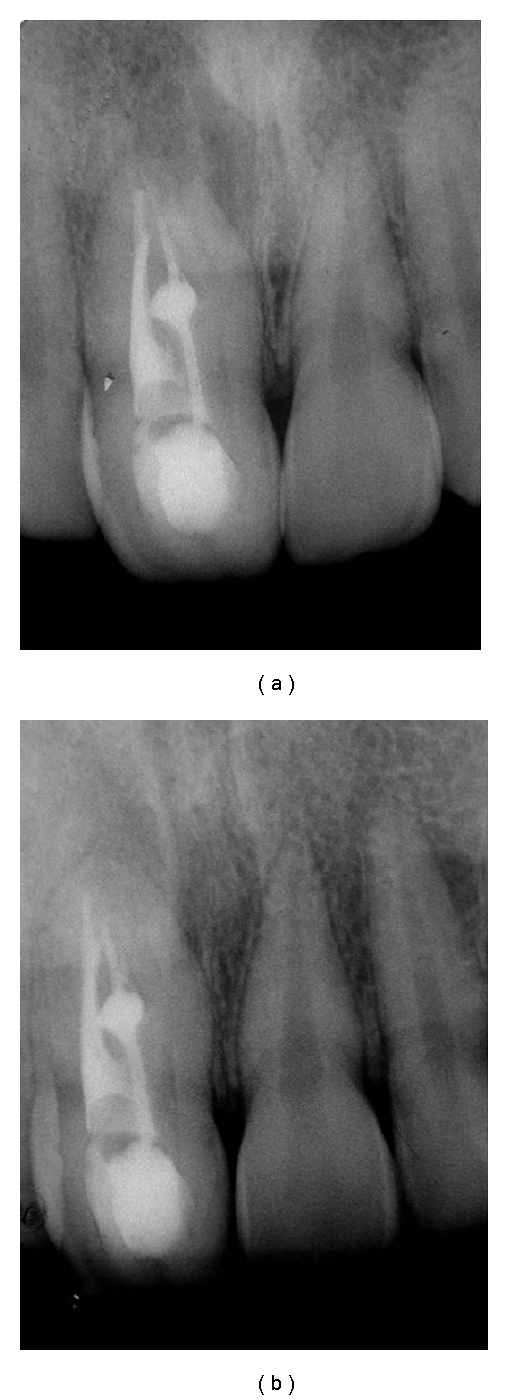
Controls of endodontic treatment. (a) One year after the conclusion of the case. (b) Follow-up period of 5 years.

A 12-year-old male with melanoderma reported to the dental service of Dentistry Faculty (University of Cuiaba, Cuiaba-MT, Brazil) with spontaneous pain in the anterior upper region. Discreet edema was observed in the apical area of tooth no. 11. A pulp vitality cold test with Endo Frost (Wilcos of Brazil, São Paulo, SP, Brazil) refrigerated gas on tooth no. 11 presented negative response. Periapical X-ray evidenced type III dens invaginatus and a radiolucid image at the apex of tooth no. 11 (Figure 1). After the absolute isolation, a conventional coronary opening was accomplished with no. 1013 diamond burr (KG Sorensen, São Paulo, SP, Brazil) and no. 3083 conic-trunk (KG Sorensen, São Paulo, SP, Brazil) allowing the observation of a brilliant flat aspect enamel structure, which was removed with a no. 1013 diamond tip (KG Sorensen, São Paulo, SP, Brazil). With the aid of a no. 2 straight tip (Maillefer-Dentisply, Baillagues, Switzerland), the presence of two canals was detected. No communication was observed between the main and invaginated canals. The two canal work lengths were established at one millimeter from the radiographic apex (Figures 2(a) and 2(b)). Biomechanical preparation was accomplished by preparing the cervical and middle thirds with Gates Glidden drills no. 1 and no. 2 (Maillefer-Dentisply, Baillagues, Switzerland), and the surgical diameter was determined with a no. 45 K file (Maillefer-Dentisply, Baillagues, Switzerland). At each instrument changing, the canals were irrigated with 2 mL of 1% sodium hypochlorite. The final irrigation was accomplished with 2 mL of 17% EDTA for 3 minutes, followed by 2 mL of 1% sodium hypochlorite. After the root canal was dried with absorbent tips (Maillefer-Dentisply, Baillagues, Switzerland), the intracanal medication composed of calcium hydroxide associated with physiologic serum was administered at 30-day intervals, for a 2-month period. The tooth was then filled through no. 55 McSpadden thermoplastizers (Maillefer-Dentisply, Baillagues, Switzerland) with zinc oxide and eugenol cement and gutta percha cones (Maillefer-Dentisply, Baillagues, Switzerland) (Figure 2(c)). The first follow-up visit was accomplished one year after case conclusion (Figure 3(a)) with a follow-up period of 5 years (Figure 3(b)).

## 3. Discussion

Dens invaginatus constitutes a challenge to endodontic treatment, due to its complicated root canal system. In types I and II, the invagination can be removed, thus transforming the tooth into a single canal followed by conventional treatment [23]. The challenge becomes greater in type III cases, where the anatomy is more complex [26, 42]. Although surgical treatment is an option, nonsurgical endodontic treatments have recently been reported [7, 18, 27–29]. Extraction is indicated only in those cases, where endodontic therapy and parendodontic surgeries failed or were not possible [30]. The present case shows the occurrence of type III dens invaginatus in tooth no. 11 with periapical lesion, which was properly treated through an orthograde procedure.

Teeth with invagination are more susceptible to carious lesions as a consequence of the pulpal topography that serves as retention material, as well as structural defects at these areas, where the enamel is badly formed or is not present [7, 13, 26–30, 40]. Numerous thin canals allow communication with the pulp, making it possible for microorganisms and their products to reach the pulp, leading to pulpal infection and necrosis [39], as in the present case. Therefore, the invagination of type III dens invaginatus often has communication with the oral cavity, allowing irritants and microorganisms to enter directly into the pulpal cavity including the area that is separated from the pulpal tissue by a thin enamel layer and dentine [26]. This condition commonly leads to necrosis of the adjacent pulpal tissue and to the development of periapical lesions soon after the tooth eruption [42–44]. However, in cases of early clinical or radiographic diagnosis of invagination without signs of pulp pathology, fissure sealing, and restorations can effectively be accomplished [6].

Considering the clinical progression of type III dens invaginatus, some aspects should be reported. In the present case, after finishing biomechanical preparation and abundant irrigation with 1% sodium hypochlorite, calcium hydroxide with physiologic serum was used as intracanal medication [34, 45, 46]. Taking into consideration the necessity for fast liberation of calcium ions [47], an aqueous medium was used.

Another important aspect is the dens invaginatus filling, which due to the enamel invagination presents a wide and bulky cavity, requiring an obturation with filling material. The thermoplasticizing techniques can facilitate the procedure and provide a more efficient sealing [7], as accomplished in the present study.

Radiographic and clinical five-year followups were responsible for the success in this case, demonstrating that conventional endodontic treatment through orthograde techniques is useful in cases of type III dens invaginatus. This finding is in agreement with previous clinical reports [30, 32].
